# Editorial: Emerging trends in abdominal and pelvic pain

**DOI:** 10.3389/fpain.2023.1254682

**Published:** 2023-08-30

**Authors:** Bin Feng, Anna P. Malykhina

**Affiliations:** ^1^ Department of Biomedical Engineering, University of Connecticut, Storrs, CT, United States; ^2^ Department of Surgery, University of Colorado Anschutz Medical Campus, Aurora, CO, United States

**Keywords:** abdominal pain, visceral pain, colorectal distension, endometriosis, dysmenorrhea

**Editorial on the Research Topic** Emerging trends in abdominal and pelvic pain

Abdominal pain is a cardinal symptom of many disorders of the lower urinary, gastrointestinal and reproductive tracts that requires effective therapeutic management. Pathological changes in one of the pelvic viscera may significantly affect the function/sensation/pain in an adjacent organ, and disrupt normal physiological coordination between the organs (1). The development and persistence of chronic abdominal and pelvic pain is associated with long-lasting modifications in neural, endocrine, paracrine and immune mechanisms supporting daily physiological activity of the involved organs (2). Existence of cross-innervation, functional cross-talk between pelvic organs, as well as involvement of peripheral and central sensitization in chronic pain makes it difficult to identify the original source of pain in the clinic (3). Extensive research has been devoted to understanding the mechanisms of prevalent pain-related conditions, and is necessary to advance the development of novel therapeutic approaches to reduce the impact of abdominal pain on patients' daily lives (4).

Dysmenorrhea, a condition characterized by painful menstrual cramps in women, causes a significant decline in productivity and quality of life. Two recent studies have investigated the underlying mechanisms of dysmenorrhea, examining the role of intestinal gas and catastrophizing as potential contributing factors (Zhang et al.; Redwood et al.). Additional research methods, such as magnetic resonance imaging (MRI) for measuring colonic volume and specialized questionnaires for dysmenorrhea catastrophizing, have been implemented. Due to the adverse effects of opioid-based pain management, complementary and integrative methods like electrical neuromodulation have gained more acceptance among doctors and chronic pain patients. Studies of the mechanisms and signaling pathways of neuromodulation using pre-clinical animal models will help further advance the clinical efficacy of neuromodulation. To that end, Zhang et al. aimed to test a new anesthesia protocol for assessing visceral pain in mice and evaluating the effectiveness of invasive neuromodulation. Additionally, a perspective article by Redwood et al. evaluated aged-garlic extract (AGE) as a non-opioid prophylactic treatment for endometrial pain.

The study titled “Menstrual Cycle Variation in MRI-Based Quantification of Intraluminal Gas in Women With and Without Dysmenorrhea” by Oh et al. explored the potential contribution of intraluminal gas to dysmenorrheal symptoms. Using MRI, the authors measured colonic gas volumes in women with and without dysmenorrhea. Common symptoms during the perimenstrual period included abdominal pain, bloating, and flatulence. The findings revealed that menstrual cycle variations did not directly influence gas accumulation, as there were no significant differences in intraluminal gas volume between different phases of the menstrual cycle. Additionally, women with dysmenorrhea had less intraluminal gas during menstruation compared to pain-free participants. The obtained results challenge the hypothesis that increased intraluminal gas volume can contribute to abdominal symptoms during menses. Other factors like alterations in the intestinal microbiome or increased visceral sensitivity also may play a role in dysmenorrheal pain. These findings emphasize the need for further investigation into the complex mechanisms of abdominal symptoms during menstruation.

The manuscript titled “Dysmenorrhea Catastrophizing and Functional Impairment in Female Pelvic Pain” explored the relationship between dysmenorrhea catastrophizing and pain interference in women with chronic pelvic pain (CPP) (Li et al.). The study involved 104 women with CPP who completed several questionnaires to assess dysmenorrhea catastrophizing, pain interference, and other related factors. The results revealed a significant association between dysmenorrhea catastrophizing, as well as dysmenorrhea interference and CPP-associated pain interference. The intensity of dysmenorrhea was identified as the most important predictor of catastrophizing. These findings highlight the importance of addressing dysmenorrhea catastrophizing to mitigate the functional impact of the condition, and to improve patients' quality of life. Effective management of dysmenorrhea symptoms to reduce pain intensity is crucial in alleviating interference caused by the condition (2). Furthermore, targeting dysmenorrhea catastrophizing early on may prevent or reduce the severity of future CPP. This research fills a significant gap in understanding the role of dysmenorrhea-specific catastrophizing in pelvic pain interference, and emphasizes the consideration of cognitive and emotional responses in developing novel interventions to treat female pelvic pain.

Managing visceral pain associated with irritable bowel syndrome (IBS) remains a clinical challenge. Objective biomarkers are crucial for early diagnosis and development of effective treatments. Colorectal distension (CRD) is one of the validated approaches to assess visceral sensitivity. However, measuring visceromotor responses (VMR) in response to CRD in awake rodents is hindered by movement artifacts, thereby, limiting its effectiveness for testing pain management strategies. To overcome these limitations, Zhang et al. tested an optimized anesthesia protocol for reliable VMR recordings during CRD in deeply anesthetized mice (Zhang et al.). The researchers employed a two-hour window with prolonged urethane infusion followed by surgical procedures under 2% isoflurane anesthesia. The surgical protocol included suturing wire electrodes to the abdominal muscles, placing a polyethylene catheter for urethane infusion, and inserting a plastic-film balloon intra-anally. The mice were then switched to the new urethane anesthesia protocol, involving an initial infusion and continuous low-dose infusion throughout the experiment. The study investigated the correlation between balloon depth on VMR to CRD, and observed depth-dependent responses. Additionally, VMR was assessed in mice with TNBS-induced transient colonic inflammation, revealing a previously unreported sex difference (Zhang et al.). The optimized anesthesia protocol provided a valuable tool with improved consistency of VMR recordings, allowing objective assessment of various neuromodulatory strategies to alleviate visceral and pelvic pain.

Endometriosis, a debilitating CPP condition with limited treatment options, may benefit from aged-garlic extract (AGE), according to a recent study by Redwood et al.. AGE contains biologically active compounds with immunomodulatory and antioxidant properties, making it a potential prophylactic option for managing endometriosis-related pain. Endometriosis involves the growth of uterine tissue outside of the uterus, causing chronic pelvic pain and reduced quality of life. Current treatments offer temporary relief or have unwanted side effects. Garlic, known for its anti-inflammatory and antioxidant effects, has demonstrated some promise in reducing endometrial pain. Human trials previously showed a pain reduction through decreased oxidative stress and endometrial cell proliferation. Garlic extracts inhibit the proliferation of endometrial stromal cells, and reduce the expression of adhesion molecules. Therefore, garlic's antioxidant properties may counteract inflammation-induced oxidative stress. N-acetylcysteine (NAC), found in garlic, exhibits anti-proliferative effects on endometriomas and downregulates inflammatory pathways. AGE, in particular, contains concentrated immunomodulatory and antioxidant compounds that can benefit endometriosis management. Further research and clinical trials are necessary to validate its effectiveness in managing endometriosis and alleviating CPP.

In summary, the diagnosis of pelvic pain and respective co-morbidities requires careful investigation of all pelvic organs that could potentially contribute to CCP. Clinical “endo-phenotyping” is a way to identify and group patients based on the trigger points, pain profiles and possible susceptibility to a certain treatment. The development and use of translational animal models, which closely mimic clinical conditions is beneficial to study the mechanisms of pelvic pain, and to identify potential biomarkers for CPP disorders. The latest technological advances including machine learning and artificial intelligence should be tested and incorporated in the development of automatic pain assessments for future use in the pain clinics and clinical research studies (5). Overall, clinical investigations of patients and research on human subjects should be more closely integrated with animal models to improve the diagnosis and treatment of abdominal and pelvic pain, making integrative and interactive research a top priority in the CPP field.

## References

[1] Malykhina AP. Neural mechanisms of pelvic organ cross-sensitization. Neuroscience. (2007) 149:660–72. 10.1016/j.neuroscience.2007.07.053 17920206

[2] Juganavar A Joshi KS. Chronic pelvic pain: a comprehensive review. Cureus. (2022) 14:e30691. 10.7759/cureus.30691 36465795 PMC9709590

[3] Pichetshote N Pimentel M. An approach to the patient with chronic undiagnosed abdominal pain. Am J Gastroenterol. (2019) 114:726–32. 10.14309/ajg.0000000000000130 30694864

[4] Chen L Ilham SJ Feng B. Pharmacological approach for managing pain in irritable bowel syndrome: a review article. Anesth Pain Med. (2017) 7:e42747. 10.5812/aapm.42747 28824858 PMC5556397

[5] Cascella M Schiavo D Cuomo A Ottaiano A Perri F Patrone R Artificial intelligence for automatic pain assessment: research methods and perspectives. Pain Res Manag. (2023) 2023:6018736. 10.1155/2023/6018736 37416623 PMC10322534

